# Positive Immuno-Modulation Following Radiofrequency Assisted Liver Resection in Hepatocellular Carcinoma

**DOI:** 10.3390/jcm8030385

**Published:** 2019-03-19

**Authors:** Kai Wen Huang, Kumar Jayant, Po-Huang Lee, Po-chih Yang, Chih-Yang Hsiao, Nagy Habib, Mikael H. Sodergren

**Affiliations:** 1 Department of Surgery & Hepatitis Research Center, National Taiwan University Hospital, Taipei 10051, Taiwan; skyntuh@gmail.com (K.W.H.); pohuang111@ntu.edu.tw (P.-H.L.); cyhsiao1102@gmail.com (C.-Y.H.); 2 Centre of Mini-invasive Interventional Oncology, National Taiwan University Hospital, Taipei 10051, Taiwan; 3 Department of Surgery and Cancer, Imperial College London, London SW7 2AZ, UK; nagy.habib@imperial.ac.uk (N.H.); m.sodergren@imperial.ac.uk (M.H.S.); 4 Department of Renal Transplant Surgery, Royal Free Hospital, London NW3 2QG, UK; 5 Warwick Medical School, University of Warwick, Coventry CV4 7HL, UK; 6 Graduate Institute of Clinical Medicine, College of Medicine, National Taiwan University, Taipei 10051, Taiwan; youngbirds@gmail.com; 7 Center for Organ Transplantation and Liver Disease Treatment, Fu Jen Catholic University Hospital, New Taipei City 24352, Taiwan; 8 School of Medicine, Fu Jen Catholic University, New Taipei City 24352, Taiwan

**Keywords:** liver cancer, radiofrequency based device, liver resection

## Abstract

Introduction: Hepatocellular carcinoma (HCC) often develops on a background of chronic inflammation and a complex immunosuppressive network with increased regulatory T cells, impaired CD8^+^ T cells and the secretion of immunosuppressive cytokines. Previous clinical studies have reported a superior disease-free survival (DFS) following a radiofrequency-based ablation or resection in HCC tumours compared to conventional liver resection techniques. The aim of this study was to investigate whether there is any correlation with the use of a radiofrequency-assisted liver resection and clinical outcome. Material and Methods: Patients’ peripheral blood was collected prior and 7 days following surgery from patients undergoing a liver resection for HCC. There were 5 liver resections performed using CUSA and 6 liver resections with the RF-based device, Habib^TM^ 4X. The primary endpoint of the study was to assess the immunological parameters of circulating immune cell populations as well as serum cytokines. The Student’s *t*-test, chi-square or Fisher’s Exact test were applied for statistical comparisons, as appropriate. Results: Patients undergoing an RF-assisted liver resection with Habib^TM^ 4X had a significant decrease in the inhibitory Treg cells (*p* = 0.002) and a significant increase in CD8^+^ T lymphocytes (*p* = 0.050) and CD4^+^CD45RO^+^/CD4^+^ memory T cells (*p* = 0.002) compared to those patients undergoing a liver resection with CUSA. It was also noted that the RF-assisted liver resection group had a significant decrease in circulating TGF-ß (*p* = 0.000), IL10 (*p* = 0.000) and a significant increase in IFN-gamma (*p* = 0. 027) and IL-17 compared to the CUSA group. Conclusion: A liver resection with RF-based device Habib^TM^ 4X was associated with positive immunomodulatory changes in circulating immune cells and circulating cytokines which could explain the significant improvement in DFS.

## 1. Introduction

Hepatocellular carcinoma (HCC) is a primary tumour associated with increasing incidence and mortality [1,2]. The Surveillance, Epidemiology and End Results (SEER) Database of the National Cancer Institute in the States, outlined a ≈3% annual increase in the HCC incidence during the period of 2008–2012 and a 3% increased annual mortality [3,4]. The contemporary advancement in the surgical and non-surgical techniques such as radiofrequency ablation (RFA), trans-arterial chemoembolization (TACE), chemotherapy, liver resection, and liver transplantation have brought a significant impact on the management of patients with liver cancer [5,6]. A considerable amount of vexation observed in the management of HCC is mainly determined by the cancer stage and feasible treatment options available at that stage. In the contemporary world, percutaneous local ablations such as RFA, liver resection and transplantation are recommended therapies of curative intent in patients presenting with early stage primary liver tumours (<3 cm) with an observed 5-year survival of 50–75%; whilst patients with HCC tumours of size >3 cm have a median overall survival of 16 to 22 months [7,8,9]. Hence, the observed clinical outcomes are the consequence of the distant micro metastasis observed with HCC, and tumours often escape the loco regional destruction offered by conventional surgical resection(s) [10]. The tumour biology is a systematic concept according to which the behaviour of cancer is not only determined by the genetics of tumour cells, but also by the microenvironment. The tumour cells escape immunological surveillance by diminished recognition by immune cells through CD8^+^, CD4^+^ T cells and natural killer (NK) cells; the increased resistance by tumour cells; or the instigation of an immunosuppressive microenvironment via regulatory T cells (Tregs) and cytokines. An increased recurrence and metastatic dissemination in HCC patients during a post-surgery period further attests that, even though the systemic antitumor immunity is discernible in cancer patients, the steady-state immune response is ineffectual for delivering reasonable tumour control [11,12].

In accordance with the immunoediting hypothesis for the cancer development and progression, the immune system favours tumour cells which are less immunogenic or release immunosuppressive factors. The immune system eludes an anti-tumour response; in addition, by the time HCC tumours become apparent they have already unfolded several other getaways of immunological recognition and elimination [13,14,15]. Notably, three aspects of tumour biology are considered main line determinants for eluding the tumours cells from the immune system, including the abatement in the expression of tumour associated antigen (TAA) and major histocompatibility complex (MHC) class I, leading to the debacle of CD8^+^ T cells to discern cancer cells; immune checkpoint inhibitions that accrue myriads of immunosuppressive molecules, including cytotoxic T lymphocytes-associated antigen-4 (CTLA-4 or CD152), programmed death 1 (PD-1) or its ligand PD-L1, T cell membrane protein 3 (TIM-3), killer cell immunoglobulin-like receptors (KIR), and lymphocyte activation gene 3 (LAG-3); and the induction and infiltration of immunosuppressive cells like regulatory T cells (Tregs), myeloid-derived suppressor cells (MDSCs) and tumour-associated macrophages to stifle the anti-tumour immune response [16,17].

HCC eludes the anti-tumour immunity by fostering an intricate network of immunosuppression pathways involving tumour and stromal cells by instigating a response opposing the priming of T cells and immune effector functions through the secretion of multitude immunosuppressive cytokines such as IL-10, IFN-γ, TGF-β, IDO, and so on [18]. Moreover, the constraints of immunosuppressive forces and the constant exposure to tumour antigens result in T-cells exhaustion, a process partially conciliated through the intramolecular expression of immune-inhibitory factors [19,20]. One potential way to overcome the challenge of the tumour microenvironment is to induce and augment the systemic antitumor immunity by activating the body immune system [21,22]. The energies used in various thermal ablative techniques such as cryoablation, radiofrequency (RF), microwave, and focused ultrasound (FUS), have the potential to trigger an anti-tumour immune response, which can minimize the tumour recurrence risk by eliminating micrometastatic residual disease [23,24,25]. The ablation of HCC nodules give rise to tumour antigens as an in-situ cancer vaccine, which can lead to the initiation of a systemic antitumor immune response that can potentially eliminate occult, metastatic tumours. The phenomenon of activation of the immune system with a distal antitumor response is known as the abscopal effect [26]. The RF energy delivered through radiofrequency based (RF-based) devices initiates ionic agitation and generates high-temperature focal hyperthermia (150 °C), thereby producing irreparable cellular damage and coagulative necrosis. In addition to the cytoreductive antitumor activity, various preclinical and clinical studies have ascribed the potential of RF in fostering an anti-tumour immune response by virtue of its immunomodulatory effects. The debris produced following RF-induced coagulative necrosis during liver resection generates tumour antigens and chemokines, which enticed the immunoprotective infiltrates, macrophages, neutrophils, DCs, and NK cells. DCs activate the nuclear factor kappa-light-chain-enhancer of the activated B cells (NF-κβ) pathway, which stimulates CD8^+^ and CD4^+^ T lymphocytes and promote a systemic immune response also known as the “in-vivo dendritic cell vaccine effect” [27,28,29,30]. A strikingly dense CD3^+^ T cell infiltration has been demonstrated by studies at tumour locations following radiofrequency ablations (RFA), consistent with the local antitumor immune response. Further evidence of a RFA-induced systemic immunity stems from preclinical and clinical reports of abscopal effects involving the spontaneous regression of distant metastatic lesions following the ablation of primary lesions [31,32,33].

The RF-based device Habib^TM^ 4X, introduced the bloodless technique of liver resection and has transformed liver surgery for last two decades. Here, RF generated coagulative necrosis on the hepatic parenchyma creates an avascular plane for resection adjacent to the tumour mass. Most HCC resections are completed on livers with an underlying cirrhosis or fibrosis that diminishes the hepatic functional reserve and heightens the likelihood of a hepatocellular failure. Consequently, resections have to be as conservative as achievable in the ablation of non-tumoural hepatic parenchyma. [30,34,35,36]. Qiu et al. (2017) [37] have outlined the benefits of a liver resection with Habib^TM^ 4X and demonstrated significantly less morbidity, mortality and better survival than with the conventional clamp-crush technique.

In our previously published study (2017) [34], we compared the oncological outcomes following a liver resection in HCC patients using an RF-based device Habib^TM^ 4X with a cavitron ultrasonic surgical aspirator (CUSA) and reported a significantly longer disease-free survival in the Habib 4X group (50.80 vs. 45.87 months, *p* = 0.03). Herein, we are presenting the immunomodulatory changes in the HCC patients, following a liver resection in HCC patients using an RF-based device Habib^TM^ 4X with CUSA, which are based on the fact that anti-tumour immune responses following radiofrequency applications in HCC tumours mark better oncological outcomes.

## 2. Experimental Section

### 2.1. Study Design

We prospectively analysed the data from two centers of National Taiwan University Hospital following the approval from the Institutional Review Board. The data included 11 patients with a proven diagnosis of HCC, who underwent a liver resection with a CUSA or RF based device Habib^TM^ 4X from July 2017 to May 2018. The primary endpoint of the study was to assess pre- and post-liver resection immunological parameters: circulating cell populations and serum cytokines.

### 2.2. Subjects and Procedures

A total of 11 patients with HCC were included in this study, 5 liver resections were performed using CUSA whilst the RF-based device Habib^TM^ 4X was the modality of choice in 6 patients. An open surgical hepatectomy was completed under the guidance of an intra-operative ultrasound. In this study, the resection of three or more liver segments was considered a major hepatectomy whilst fewer than that was considered a minor hepatectomy. Both the lobes of the liver were mobilized and, if needed, the gall bladder was removed. Inflow control was sought in selective cases where excessive parenchymal bleeding was envisaged. In situations where a hepatic parenchymal resection was accomplished with CUSA, an additional help from an assistant surgeon was required to curb the risk of haemorrhage utilizing bipolar coagulation; however, no assisting haemostatic device was obligated to perform such a task in the Habib^TM^ 4X group. An RF-based bipolar device was applied perpendicularly onto the hepatic parenchyma in a sequential manner to create parallel lines of ablation. An additional line of ablation was fashioned in a perpendicular manner to join the parallel track. Throughout, the application probe was moved in and out in a sequential fashion for 3–5 mm along its axis, which helped in warding off the adherence of the liver tissue. Once a 1 cm thick area of ablated and coagulated tumour free margin was achieved, the hepatic parenchyma was transected using a surgical scalpel [35,36,38]. A haemostasis was attained and the raw surface was covered with a haemostatic agent.

### 2.3. Cellular Subsets

Peripheral blood samples were collected from each patient in an EDTA anticoagulant-treated tube on day 0 (pre liver resection) and day 7 following the tumour resection. The immunophenotypic analysis was accomplished within 24 h of the sample collections.

Panel 1: Treg cells, CD8^+^, CD4^+^, CD3^+^, CD4^+^CD45RO^+^/CD4^+^, CD4^+^CD39^+^/CD4^+^, NK, NKT cells;
Panel 2: IFN-γ, TGF-α, TGF-β, IL-1b, IL-6, IL-17, IL-10.

#### 2.3.1. Lymphocytes Isolation

The 20 mL of blood were collected 7 days following the liver resection through a central venous catheter. To isolate the immunocyte, buffy coats were collected and then separated on a Ficoll-Hypaque gradient and used for further analysis.

#### 2.3.2. Flow Cytometry

The cells were processed, brought to single cell suspensions in PBS with 0.5% BSA. and stain at 4 °C for 30 min. The cell surface markers were stained with fluorescent-labeled antibodies: FITC-CD45, anti-CD39-FITC, PE-CD8, PerCP-CD3, anti-CD45RO-ECD, anti-CD45RA-ECD, CD161-DX12, APC-CD25, PE-CD127 and APC.Cy7-CD4 from BD Biosciences (San Jose, CA, USA), CD4^+^CD45RO^+^ cells are considered an activated and short-life memory helper T cell subset.

The cells were then washed twice and fixed by fixation buffer (BD Biosciences, San Jose, CA, USA). The total numbers of individual leukocyte subsets were determined using 123count eBeads counting beads (eBioscience, San Diego CA, USA). A flow cytometry was performed by FACSVerseTM (Becton Dickinson, Mountain View, CA, USA), and the data were processed using FlowJoTM software (Ashland, OR, USA).

#### 2.3.3. Data Analysis and Absolute Count Determination

(1) Use normal gating strategies to identify the cell population to be enumerated (i.e., FSC/SSC lymphocyte gate CD3^+^CD4^+^ gate);
(2) In the same sample, draw a gate on 123 count eBeads in an ungated plot displaying two blue (488 nm) or violet (405 nm) laser excited parameters;
(3) Using the count statistics from these two gates, the concentration of the original cell sample may be determined by the equations:
  Absolute cell number (cells/μL) = (cell count × eBead volume)/(eBead count × cell volume) × eBead concentration (1000/μL)

#### 2.3.4. Serum Assay

Circulating immunoreactive IFN-γ, TGF-α, TGF-β, IL-1b, IL-6, IL-17 and IL-10 levels were measured using commercially available quantitative enzyme-linked immunosorbent assays (ELISA, R&D Systems Europe, Abingdon, UK). The assays did not measure the biological activity of the cytokines. All the measurements were made by a single trained individual to avoid any interobserver variation. All the samples were assayed in duplicate to ensure accuracy and validity.

### 2.4. Statistical Data Analysis

All the data were entered into a Microsoft Excel™ database and analysed using SPSS_24.0 software (version 24, IBM, Armonk, NY, USA). Continuous variables were analyzed with a Student’s *t*-test, and categorical variables were analyzed with a chi-square or Fisher’s Exact Test where appropriate. Furthermore, the paired Student’s *t*-test were used to compare data between pre- and post-liver resection immunomodulatory changes in the respective groups. *p* values under 0.05 were considered statistically significant.

### 2.5. Ethical Approval

All procedures performed in studies involving human participants were in accordance with the ethical standards of the institutional and/or national research committee and with the 1964 Helsinki declaration and its later amendments or comparable ethical standards.

## 3. Results

### 3.1. Demography

A hepatic resection was performed in 11 HCC patients, of which a CUSA-based resection was accomplished in 5 patients, whilst in 6 patients a resection was performed using the RF-based device Habib^TM^ 4X. The demographic parameters for each group are outlined in Table 1. The mean age of patients in the CUSA and Habib^TM^ 4X group was 66.00 ± 17.00 years and 62.00 ± 12.80 years respectively. There were 4 women (80.0%) and 1 man (20.0%) in the CUSA cohort, and 1 woman (16.66%) and 5 (83.3%) men in the Habib-4X group. Along with that, we didn’t observe any significant differences between the groups regarding serum albumin, serum bilirubin, serum AFP, tumour numbers, tumour size, tumour stage, cirrhosis, HBsAg (hepatitis B surface antigen), HCV (hepatitis C virus), ICG (indocyanine green) clearances and tumour characteristics (Table 1 and Table 2).

### 3.2. Pre- and Post-Liver Resection Modulation of Circulating Immune Cells

**Figure 1:**
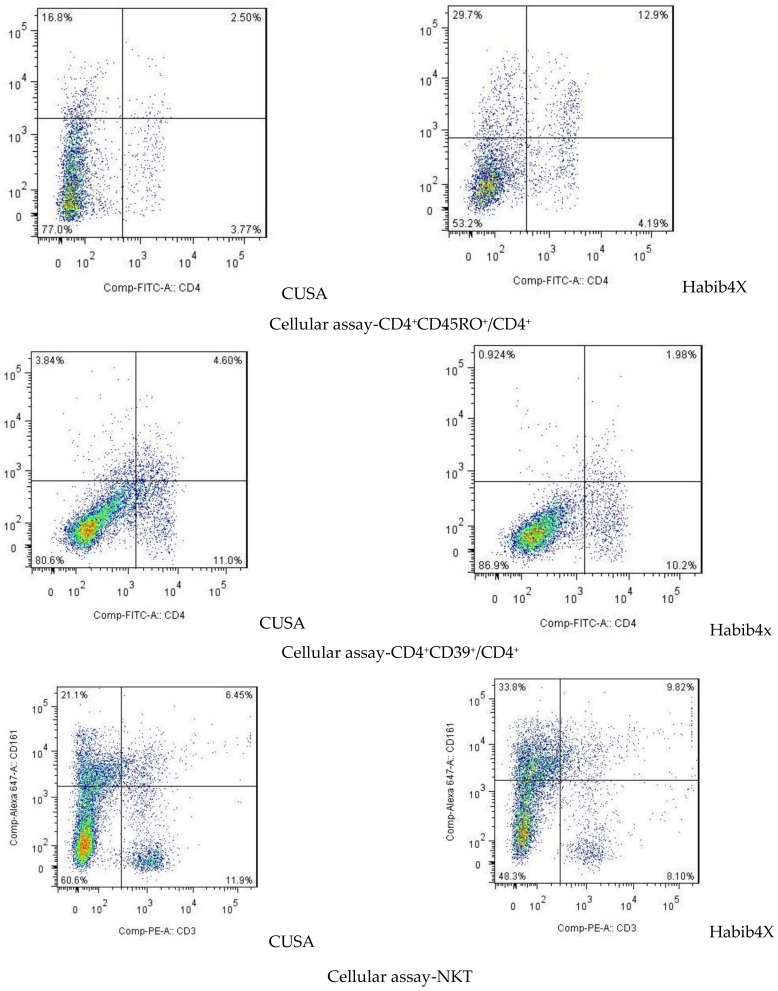
Flow cytometric analysis of immune cells in peripheral blood 7 days after liver resection with CUSA or Habib^TM^-4X.

We evaluated the absolute number of several immune cell populations i.e., cytotoxic T cells (CD8 T cells), helper T cells (CD4 T cells), regulatory T cells (Treg cells), and natural killer (NK) T cells (Table 3; Figure 1).

**Figure 2:**
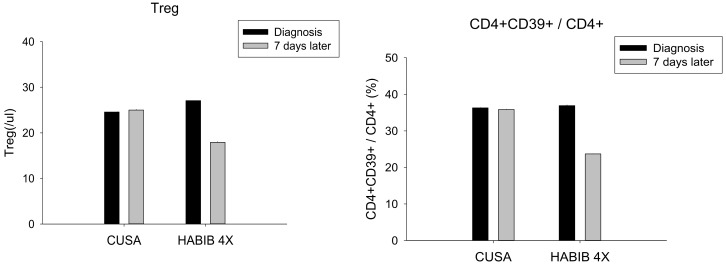
Treg cells and the CD4^+^CD39^+^/CD4^+^ cells changes in both study groups. A significant decrease was observed in both cell types in the Habib^TM^ 4X group.

**Figure 3:**
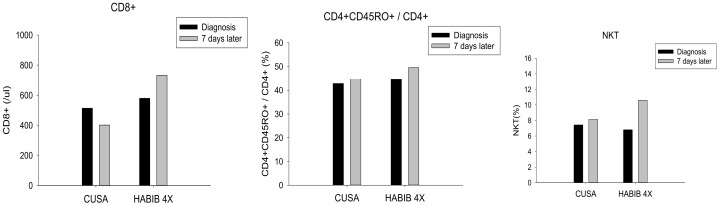
Cytotoxic CD8 T cells, CD4^+^CD45RO^+^/CD4^+^ and NKT cells changes in both study groups. A significant increase was observed in all three cell types in the Habib^TM^ 4X group.

The data demonstrated a significant decrease in Treg cells (*p*-value = 0.002) and CD4^+^CD39^+^/CD4^+^ cells (*p*-value = 0.002) following surgery in the Habib^TM^ 4X group whilst no such observation was made in the CUSA group (Figure 2). Furthermore, our study reported a significant rebound in CD8^+^ (*p*-value = 0.050), CD4^+^ CD45RO^+^/CD4^+^ (*p*-value = 0.002) and NKT cells (*p*-value = 0.002) after the liver resection in the Habib^TM^ 4X group whilst no such modulation was noted in the CUSA group (Figure 3). On the contrary, NK cells and CD4^+^ cells alone were not significantly modulated after the resection in any of the study group.

The data of interest in the present immunological analysis was pertinent to two main T cell subpopulations: the Treg cells and the cytotoxic CD8 T cells. Both subsets showed significant alterations following the liver resection with an RF-based device Habib^TM^ 4X, suggesting the activation of the adaptive immune response. Moreover, the study exhibited a considerable decrease in immunosuppressive Treg cells which play a crucial role in tumour growth and metastasis; hence, the decline in these subsets of cells following the liver resection in Habib^TM^ 4X group confirms the ability of RF to promote the systemic immune response.

### 3.3. Pre- and Post-Liver Resection Modulation of Circulating Cytokines and Chemokines

We analysed the plasma concentration of several metabolites such as cytokines, interleukins and chemokines able to modulate the immune response, at the same time points in which we tested the blood immune cell composition. The study demonstrated that the RF based device Habib^TM^ 4X was associated with marked changes in the plasma concentration of IFN-γ, TGF-ß, interleukin (IL)-10, and IL-17 (Table 2).

**Figure 4:**
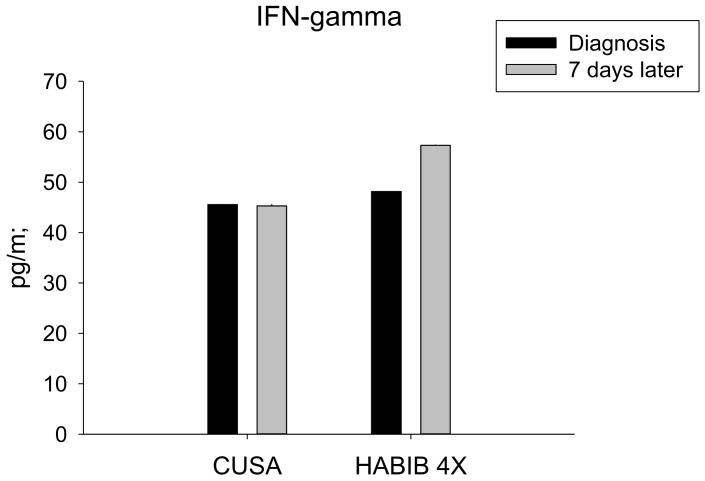
Serum IFN-γ changes in both study groups. A significant increase was noted in the Habib^TM^ 4X group.

The serum IFN-γ level was significantly increased in the Habib^TM^ 4X group in comparison to CUSA (*p*-value = 0.027), as lower serum levels of IFN-γ were associated with increased Tregs and marked tumour growth and progression (Figure 4).

**Figure 5:**
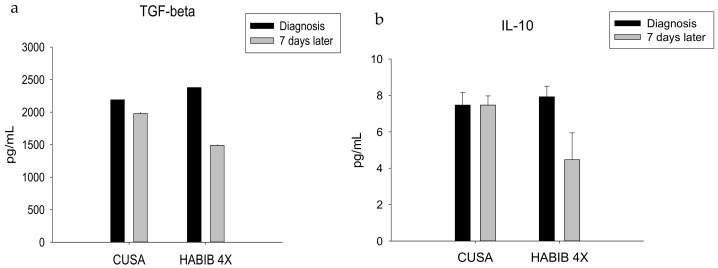
Serum TGF-ß (**a**) and IL-10 (**b**) level changes in both study groups. Significant decrease was noted in Habib^TM^ 4X group.

The RF-based device Habib^TM^ 4X did significantly decrease in TGF-ß (*p*-value = 0.002), which normally favours unregulated tumour-growth by sustaining cancer angiogenesis and enhanced tumour associated inflammation (Figure 5a). Similarly, we noted a decline in IL-10 following the resection with the RF-based device Habib^TM^ 4X, which not only directly suppresses cytotoxic T-cells and NK cells but also promotes tumour progression and a poor prognosis (Figure 5b). Both TGF-ß and IL-10 played an instrumental role in the induction of Treg cells and the abolition of NKT cell activity.

**Figure 6:**
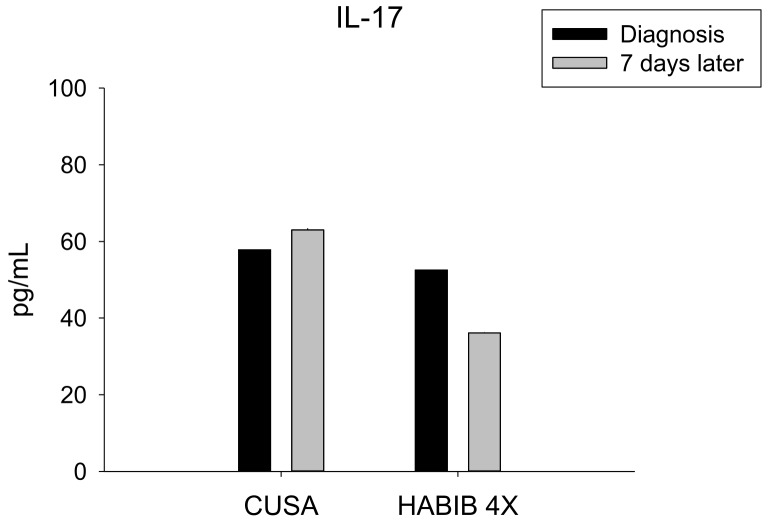
Serum IL-17 level changes in both study groups. Significant decrease was noted in Habib^TM^ 4X group.

IL-17 constituted a crucial component of the inflammatory background of HCC, and a high expression was considered as a predictor for disease progression and poor survival. The data analysis outlined a significant decrease in IL-17 following the RF-based liver resection, in contrast to CUSA (*p*-value = 0.010) (Figure 6).

In addition, we analyzed a few other metabolites, including TGF-α and IL-1b; however, we could not find any significant changes in their levels.

## 4. Discussion

In acts of deception, HCC evades the natural anti-tumour immunity through the formation of an extremely intricate immunosuppressive network. The interactions between the malignant cells with immune and stromal cells instigate the secretion of various immunosuppressive cytokines. The malignant cells of HCC use autonomous and non-autonomous techniques to escape the body’s inherent anti-tumour immune response. The selective pressure on transformed cells activates a phenomenon of immunoediting in the immune system, which comes to the aid of cells with less immunogenic potential or who produce immunosuppressive factors. It is reckoned that tumour cells and a multitude of components in the tumour microenvironment conspire to inveigle their own development and progression. The tumour cells circumvent the inherent immunological surveillance system by limiting the recognition by immune cells, including CD8^+^, CD4^+^ and natural killer (NKT) cells [39,40]. The silencing or repressed expression of tumour-associated antigens help tumour cells dodge the immune system despite the persistent expression of antigenic molecules owing to a glitch in antigen processing and presentation. The down-regulated expression pathway involves a proteasomal malfunction leading to a defect in protein fragmentation for the configuring of peptides, or leading to a fault in the antigen peptide transporters 1 and 2 that are involved in the transportation of the peptides to the endoplasmic reticulum to be put onto HLA class I heavy chains and presented over the cell membrane before getting recognized by CD8^+^ T cells via T-cell receptors (TCR). In addition, the mutation or deletion of beta2 microglobulin results in the complete loss of the HLA class I expression; however, both the absence or reduced expression of the HLA class I undermines tumour antigens recognition by cytotoxic T lymphocytes (CTLs) and natural killer (NK) cells [41,42,43].

The induction and infiltration of immunosuppressive cells such as regulatory T cells, myeloid-derived suppressor cells (MDSCs) and tumour-associated macrophages impede the immune response against tumour cells. Treg cells account for 5–10% of CD4^+^ T cells and are marked by the presence of the membrane molecules CD25, CTLA-4, CD62L, along with the expression of the transcription factor FoxP3, which play central roles in the maintenance of self-tolerance. FoxP3 is the key regulatory transcription factor for Treg cells, and mutations in the FoxP3 gene result in severe autoimmune disorders and the onset and progression of various cancers [44,45,46]. Studies have outlined the increased infiltration of FoxP3^+^ Treg cells in tumour and peripheral blood of HCC patients, their role in anti-tumour immunity and their aide in tumour progression. The activation of a Treg-cell through TCR engendering the inhibition of APC maturation through the CTLA-4-mediated downregulation of CD80 and CD86, repression of CD28 mediated co-stimulatory signaling, decrease of IL-2 via the enhanced expression of the IL-2 receptor with the IL-2 receptor-chain CD25, simultaneous secretion of inhibitory cytokines IL-10 and TGF-β, and ATP (adenosine triphosphate) degradation disposing the diminution of the antitumour immune response, along with the expression of granzyme and/or perforin ushered in the destruction of APCs and effector T cells [47,48,49,50]. Various studies described that tumours infiltrating Treg cells are presumed to be activated by neo-antigens released from tumour cells, are present in high concentrations within tumours and manifest the enhanced expression of suppression-related molecules such as CTLA-4. Moreover, the concentration of Treg cells reciprocates with the number of intra-tumoural macrophages and is considered as an independent negative prognostic factor for the overall survival [51]. In our study, we found a significant decrease in Treg cells and CD4^+^CD39^+^/CD4^+^ cells following the surgery with the Habib^TM^ 4X. A meta-analysis conducted by Sun et al. included 27 studies with 3854 HCC patients, and demonstrated that high intra-tumoural and peripheral blood levels of Tregs are markers of poor overall survival {OS; HR (hazard ratio) = 1.95, *p* < 0.00001} and disease free survival (DFS; HR = 1.82, *p* < 0.00001). In addition, higher Tregs infiltrations are associated with multiple liver tumours, high AFP levels, poor tumour differentiation, and advanced stage and vascular invasion, and are therefore a measure of poor prognosis [52,53].

Immune checkpoint molecules are coinhibitory in nature, and impede the immune response by steering clear of overactive T cells and averting collateral tissue damage. The important members of this group include CTLA-4, PD-1, ligands of PD-1 and TIM-3 [54,55,56,57].

CTLA-4 is constitutively expressed by Treg cells and is also expressed by activated T cells. CTLA-4 is pivotal for the control of the CD4^+^ T-cell function, and it essentially staged the priming phase of the cell mediated immune response [58,59]. It contends with the actions of the stimulatory protein CD28 by binding to its ligand CD80 and CD86 present on the membranes of APC. Moreover, CTLA-4 imparts an inhibitory signal to the T cell in opposition to conventional TCR signaling [60,61,62]. In similar fashion within the confines of the tumour, CTLA-4 fosters immunosuppression through the induction and differentiation of Treg cells along with the upregulation of IL-10 and IDO (indoleamine-2,3-dioxygenase) by means of a CD80 and CD86 counter signaling approach [63,64,65]. The upregulation of IDO in HCC is orchestrated by IFN-γ and other cytokines in HCC which inhibit T-cell activation and proliferation, and induce CD4^+^ T-cells into FoxP3^+^Treg cells. Hence, IDO also favours tumour growth by activating a path in the complex anti-tumour immunity pathophysiology [66,67]. Additionally, proinflammatory cells located in the peritumoral stroma release IL-17 and other chemokines from epithelial cells, which in turn pave the way for additional neutrophil and chemokines receptor positive B cells migrations towards the tumour. Notably, the inflammatory microenvironment produced by tumour-associated macrophages and IL-17 producing cells in HCC are not only correlated with tumour development and growth but also with recurrences following liver transplantation [68,69,70].

PD-1 is usually present on the membranes of activated CD8^+^ and CD4^+^ lymphocytes, B cells, NK cells and is also reported on MDSCs, T_reg_ cells, monocytes and dendritic cells (DCs) [54,71,72]. The expression of PD-L1 is induced by a variety of cytokines of which IFN-γ is the most potent. The tumour microenvironment is a state of chronic antigen exposure where IFN-γ released by antigen-specific T cells instigates PD-1 expression on reactive T lymphocytes and facilitates the binding with ligands (PD-L1) in APC and tumour cells. The coupling of PD-1–PD-L1 burns out T-cells by disrupting TCR signaling pathways, consequently impeding T-cell proliferation and the release of cytotoxic mediators. Additionally, colluded binding between PD-L1 (expressed on other cells) with its receptor PD-1 on macrophages causes IL-10 release and by that means CD4^+^ T-cell repression. Notably, an intense PD-1 expression on effector CD8^+^ cells within HCC tumours has been found to be related to disease progression and post-operative recurrence [73,74,75,76].

Recently, attention has been given to the inherent ability of RF, which not only kills the HCC cells but also produces favourable immunological change in the tumour microenvironment, minimizes recurrence and improves survival [77]. Studies have implicated local and systemic immunomodulatory changes following the application of radiofrequency during RFA. Consequently, the immunomodulatory properties of RF have emerged as plausible explanations for the improved survival observed in small HCC following ablation [30,78,79]. Xu et al. [80] has conducted a meta-analysis including 31 studies and 16,103 patients, and demonstrated a significantly better overall and disease-free survival of the RFA group than for conventional liver resection for small ≤2 cm HCC tumours. However logical this may seem, this theory does not hold much water owing to the underlying field change phenomenon and chronic inflammatory state of the liver over which HCCs developed. Studies have reported better survival in liver resection groups owing to the complete removal of tumour [81,82]; however logical this may seem, this theory does not hold much water owing to underlying field change phenomenon and chronic inflammatory state of the liver over which HCCs develop [83,84,85].

The immunomodulatory impact of RF in a liver resection device has never been assessed and the present study provides the first detailed analysis of such changes. Here, this study examined the pre- and post-surgery immunological parameters employing either the RF-based device Habib^TM^ 4X or CUSA for a liver resection in HCC.

Our observation demonstrated the significant advantages of immunomodulatory cellular and cytokine changes, which seem to be plausible reasons for the better survival noticed in the Habib^TM^ 4X group. RF energy induces localized coagulative necrosis during liver resection and releases a significant amount of tumour debris including immunogenic particulates, chemokines [Monocyte Chemoattractant Protein-1 (MCP-1) and CXCL16], cytokines (TNF-α, IL-1, IL-6, IL-8 & IL-16) and damage-associated molecular patterns (DAMPs), i.e., DNA and heat shock protein. These debris are taken up by DCs and presented through MHC molecules on CD8^+^ and CD4^+^ T cells to induce an immune response. The activation of the nuclear factor kappa-light-chain-enhancer of the activated B-cells (NF-κβ) pathway, stimulates CD8^+^ and CD4^+^ T lymphocytes to promote a systemic immune response called the “in-vivo dendritic cell vaccine effect” [23,29,86,87]. In the present study, we did an immune analysis of the absolute number of several immune cell populations and found significant positive changes in Treg cells, CD4^+^CD39^+^/CD4^+^, cytotoxic CD8^+^ T cells, CD4^+^ CD45RO^+^/CD4^+^ and NKT cells following the liver resection with the RF-based device Habib^TM^ 4X, in contrast to CUSA.

The T-cells infiltration on day 7 was characterised by an increase in CD8^+^ T and CD4^+^ CD45RO^+^/CD4^+^ cells, while there was a decrease in Treg and CD4^+^CD39^+^/CD4^+^ cells. This resulted in an increase in the ratio of CD8^+^ T versus Treg cells, indicating a shift of immune balance toward anti-tumour immunity following the RF application. This is in contrast to the contrary evidence, which states that surgical stress brings reduction in CD8^+^ T; this could be explained by virtue of the RF energy, which has proven to induce significant antigen specific T-cell changes in HCC [88,89,90].

Furthermore, the enhanced infiltration of CD45RO^+^ T cells with an increase in CD4^+^ CD45RO^+^/CD4^+^ cells has been considered a marker for a better clinical outcome. Hu et al. [91] performed a meta-analysis involving 25 studies and 4720 patients to understand the association between the intra-tumoural CD45RO^+^ T cells density and the overall and disease free survival in patients with HCC, and reported an improved 5-year DFS. Several mechanisms determine the immune response by CD45RO^+^ T cells in a tumour microenvironment, including a low threshold of activation upon exposure to an antigen; an enhanced capability to proliferate; an increased IFN-γ production and life-long persistence with self-renewal characteristics, all of which established them as a hallmark of adaptive immunity [92].

Tregs, especially CD4^+^CD25^+^Foxp3^+^, are one of the most studied immune cells owing to their specific inhibitory influence on HCC tumour growth and progression [93]. Zhao et al. [53] conducted a meta-analysis involving 23 studies and 1279 HCC patients to understand the association between the Tregs cells and HCC, and reported an 87% higher frequency of Tregs in the tumour microenvironment. The plausible explanations indicates towards following distinctives of Tregs cells in regulating the tumour microenvironment, including apoptosis induction of effector cells through the CD25^+^ mediated diminution of IL-2; cytolysis of effector T cells mediated by granzyme B and perforin; dendritic cells maturation and functioning regulated through CTLA-4 mediated cell-cell contact-dependent mechanism; and alteration in the effector cell immune response through the liberation of inhibitory cytokines such as TGF-β and IL-10 [94,95]. Recently, Tu et al. (2016) [96] reported a significantly poor survival in (*p* = 0.006) HCC patients with high number of intra-tumoural T-cells.

In addition, we observed a significant positive modulation in the plasma concentration of several metabolites including TGF-ß, IL-10, IFN-γ, and IL-17 following a liver resection with the RF-based device Habib^TM^ 4X, as opposed to CUSA. The secretions of these cytokines and the functioning of several immune cell populations are intricately regulated by each other; for instance, a subtype of Tregs, which play a key role in tumour immune escape, is associated with a higher secretion of IL-10 and TGF-ß and is considered a marker of progressive disease and poor survival [96,97]. TGF-β with IL-10 controls the conversion switch of type 1 and type 2 helper T cells, shifting the balance toward Type 2 helper cells. Moreover, it directly suppresses the Type 1 helper cells CD8^+^ T, NK, DC and M1-type macrophages while enhancing the expression of M2-type macrophages with the increase of Tregs cell functions [98,99]. Studies have shown that the reduction in Tregs cells would prevent the expression of immunosuppressive cytokines or that the targeted therapy against these cytokines, such as CD25, TGF-ß, CTLA-4 and so on, would prevent their functions, thereby controlling tumour growth [100]. In the present study, decreased Tregs following surgery with the RF-based device could be a possible explanation for the better survival in this group of patients, in contrast to the CUSA group, where we neither observed positive immunological changes nor better survival.

The observed immunomodulation is unique to the RF and is different from normal surgical stress or inflammation as both groups were matched in terms of age, sex, number, stages of tumours, and so on, thereby making the patients subject to an equal amount of surgical stress. In addition, the pre-liver resection immune statuses were compared with the one week post-resection statuses, which provided substantial stability in the beneficial changes of T lymphocytes and Tregs.

Furthermore, understanding the anti-tumour immunological properties of CD8^+^ T-cells and Tregs has not only led to the development of checkpoint inhibitors but also added a new dimension in the management of advanced HCC, and both CTLA-4 and PD-1 are two principles, extensively studied checkpoints, which normally prevent the overstimulation of anti-tumour immune responses. Increasing the activation of T-cell receptors and proinflammatory cytokines results in an increased CTLA-4 expression, whilst ligands of PD-1 are expressed on many immune T cells, Tregs and B-cells [101,102]. CTLA-4 attaches with costimulatory B7 molecules (CD80/86) with a stronger affinity than CD28. The binding between B7 and CTLA-4 instead of CD28, does not produce a stimulatory signal. Hence, CTLA-4 functions to competitively inhibit T cell functioning, and induce T cell anergy. However, the anti-CTLA-4 antibodies Ipilimumab and Tremelimumab have demonstrated success at overcoming this regulatory blockade. Similarly, the programmed death receptor 1 (PD-1), following activation with PD-L1, a ligand often found on tumor cells, inhibits T cell function and triggers apoptosis. Pembrolizumab, Nivolumab, Durvalumab, and Avelumab, presently available, are all anti-PD-1 drugs approved for the treatment of melanoma, Hodgkins lymphoma and various solid tumors including HCC [103,104]. Here, it is important to understand that CD8^+^ T-cells and Tregs are the centre of interest for both checkpoint inhibitors and RF; thus combining these therapeutic modalities exerts a superlative effect. Thanks to the synergism between these modalities, RF induces the infiltration of CD8^+^ T-cells at the resection margin, whilst checkpoint inhibitors enhance their anti-tumour functioning [105,106]. In accordance with this, Duffy et al. [107] conducted a study and demonstrated the activation of the immune system following the introduction of checkpoint inhibitors and the accumulation of intra-tumoral CD8^+^ T-cells following RF ablation; they thereby presented the first clinical evidence of synergism of the checkpoint inhibitor tremelimumab and RF-ablation in the management of advanced hepatocellular carcinomas. Based on our observation of positive immunomodulatory changes following liver resection with the RF-based device HabibTM 4X, we speculate that combining check-point inhibitors could improve survival or delay recurrence following resection in HCC.

The index study has certain limitations which require attention. First, secondary to the sample size and unintended biases made during the recruitment of patients could have influenced the analysis outcomes. Despite these limitations, we firmly believe that present study has analysed the broad range of circulating cell populations and serum cytokines which are involved in tumour-related immunomodulation and which could be involved in and determine the observed better survival in liver cancer patients following the application of radiofrequency energy.

In this study, we demonstrated positive immunomodulatory changes explicitly in terms of CD8^+^ T-cells and Tregs, following the liver resection in HCC patients using the RF-based device Habib^TM^ 4X, compared to CUSA, which may account for the observed better survival in the same group. The RF-based device Habib^TM^ 4X not only facilitates a safe and efficient liver resection but also fosters favourable immunomodulatory changes presumably responsible for a better survival in comparison with other modalities of liver resection. The invention of the RF-based device Habib^TM^ 4X has produced a rich array of new visions for HCC cancer treatment, focusing on the surgical resection of liver tumours with RF induced immunomodulatory changes, providing better overall and disease free survival. Further, these anti-tumour cells are a common target for RF and checkpoint inhibitors give an opportunity to combine both treatment modalities. However, future research efforts will further explore the impact of combining the checkpoint inhibitor with RF-energy during the various stages of HCC.

## 5. Conclusions

RF-based device Habib^TM^ 4X has not only commissioned a safe and bloodless hepatic resection but also persuades appreciative changes in tumour microenvironment. Henceforth, hepatic resection with RF-based device Habib^TM^ 4X in HCC are associated with positive immunomodulatory changes in circulating immune cells and cytokines which could explain the observed improvement in the DFS and decreased tumour recurrence.

## Figures and Tables

**Table 1:** Demographics and clinical characteristics of patients involved in the respective groups.

Attributes	CUSA	Habib^TM^ 4X	*p*-Value
Number of patients	5	6	NS ^#^
Mean age, Mean ± SD (years)	66.00 ± 17.00	62.00 ± 12.80	NS ^$^
No. male/female	1/4	5/1	NS ^#^
Albumin, Mean ± SD (g/dL)	4.45 ± 0.26	4.40 ± 0.59	NS ^$^
Bilirubin, Mean ± SD (mg/dL)	0.95 ± 0.60	1.04 ± 0.30	NS ^$^
Prothrombin time, Mean ± SD (sec)	11.5 ± 1.8	12.1 ± 2.10	NS ^$^
Ascites	0	0	NS ^#^
Encephalopathy	0	0	NS ^#^
ICG clearance, Mean ± SD (15 min)	7.23 ± 3.56	11.77 ± 4.04	NS ^$^
AFP ± SD (ng/mL)	79.40 ± 151.40	52.60 ± 105.30	NS ^$^
Cirrhosis	2	3	NS ^$^
HbsAg	3	1	NS ^$^
HCV	1	5	NS ^$^

AFP: alpha-fetoprotein; CUSA: cavitron ultrasonic surgical aspirator; HbsAg: hepatitis B surface antigen; HCV: hepatitis C virus; ICG: indocyanine green; NS: not applicable; SD: standard deviation. ^#^ Statistical significance was analyzed by the chi-square test. ^$^ Statistical significance was analyzed by the Student’s *t*-test.

**Table 2:** Pre and postoperative tumour characteristics of patients in study groups.

Attributes	CUSA	Habib^TM^ 4X	*p*-Value
Tumour Numbers	1–3	1–4	NS ^$^
Tumour Stage			
T1	3	4	NS ^$^
T2	2	2	NS ^$^
T3	0	0	NS ^$^
Tumour Size (cm)	3.30 ± 2.04	3.65 ± 10.60	NS ^$^
Anatomical resection	4	5	NS ^$^
Non-anatomical resection	1	1	NS ^$^
Major resection	1	1	NS ^$^
Minor resection	4	5	NS ^$^
Blood loss (mL), Mean ± SD	300.00 ± 316.00	223.00 ± 150.00	NS ^$^
Major complication	0	0	NS ^$^
Resection margin			
Free	2	2	NS ^$^
Free within 1 cm	3	4	NS ^$^
Involved	0	0	NS ^$^

AFP: alpha-fetoprotein; CUSA: cavitron ultrasonic surgical aspirator; NS: not applicable. ^#^ Statistical significance was analyzed by the chi-square test. ^$^ Statistical significance was analyzed by the Student’s *t*-test.

**Table 3:** Observed immunomodulatory changes in respective groups before and after interventions.

Parameters	CUSA			Habib^TM^ 4X		
	Before Surgery (Mean ± SD)	After 7 Days of Surgery (Mean ± SD)	*p*-Value	Before Surgery (Mean ± SD)	After 7 Days of Surgery (Mean ± SD)	*p*-Value
Treg	24.57 ± 4.83	25.00 ± 3.36	0.850	27.20 ± 6.17	17.90 ± 5.26	0.002 *
CD3^+^	1681.57 ± 384.25	1565.71 ± 459.78	0.819	1632.00 ± 392.68	1700.00 ± 445.35	0.721
CD4^+^	1085.71 ± 278.91	1095.71 ± 384.48	0.956	1008.00 ± 283.50	1028.00 ± 331.86	0.886
CD8^+^	515.71 ± 255.46	401.42 ± 98.39	0.291	580.0 ± 216.18	732.00 ± 188.31	0.050 *
CD4^+^CD45RO^+^/CD4^+^	44.71 ± 1.98	45.00 ± 4.43	0.879	44.60 ± 1.78	49.50 ± 4.03	0.002 *
CD4^+^ CD39^+^/CD4^+^	36.29 ± 4.92	35.86 ± 4.38	0.866	36.90 ± 4.23	23.70 ± 8.49	0.000 *
NK	11.86 ± 3.02	11.57 ± 3.64	0.876	11.60 ± 2.32	10.90 ± 2.51	0.526
NKT	7.43 ± 1.90	8.14 ± 2.12	0.519	6.80 ± 1.62	10.60 ± 3.50	0.006 *
TGF-ß	2191.42 ± 400.43	1978.57 ± 478.83	0.385	2378.00 ± 382.35	1490.00 ± 366.60	0.000 *
IFN-γ	45.57 ± 9.65	45.28 ± 10.73	0.959	48.20 ± 11.82	57.30 ± 7.41	0.027 *
IL-10	7.47 ± 0.69	7.47 ± 0.50	1.000	7.93 ± 0.58	4.47 ± 1.47	0.000 *
IL-1b	7.92 ± 1.47	7.90 ± 1.05	0.970	7.28 ± 1.69	9.39 ± 4.51	0.180
IL-17	58.00 ± 16.54	63.00 ± 15.35	0.569	52.6 ± 13.92	36.10 ± 13.55	0.010 *

Statistical significance was analyzed by the paired Student’s *t*-test in all scenarios. CD: cluster of differentiation; IFN-γ: interferon gamma; IL: interleukin; TGF-β: Transforming growth factor beta; Treg: T regulatory cells. * denotes statistical significance.
